# Validation of clinical simulation scenarios for the teaching of soft skills in child-centered care

**DOI:** 10.1186/s12909-024-05284-7

**Published:** 2024-03-29

**Authors:** Vivianne Izabelle de Araújo Baptista, Liliane Pereira Braga, Ádala Nayana de Sousa Mata, Bruno Oliveira Carreiro, Luiz Paulo Gomes dos Santos Rosa, Hécio Henrique Araújo de Morais, George Dantas de Azevedo, Simone Appenzeller

**Affiliations:** 1 Multicampi School of Medical Sciences of Rio Grande Do Norte, Federal University of Rio Grande Do Norte, Rio Grande Do Norte, Caicó, Brazil; 2 Graduate Program in Child and Adolescent Health, School of Medical Sciences, University of Campinas, Campinas, São Paulo, Brazil; 3 State University of Rio Grande Do Norte, Rio Grande Do Norte, Caicó, Brazil; 4 Department of Orthopedics, Rheumatology and Traumatology, School of Medical Sciences, University of Campinas, Campinas, São Paulo, Brazil

**Keywords:** Validation study, Simulation training, Clinical competence, Child health, Education, Medical, Undergraduate, Students, Medical

## Abstract

Consultations with children and their families are complex and require soft skills. However, there is a gap in the medical curriculum concerning these skills, especially as encounter training is often adult-centered. We developed, validated, and applied simulation scenarios that prioritize active participation of children to train soft skills in child-centered care for undergraduate medical students. This is a methodological study to develop three scenarios and a checklist of what is expected. The content was validated by 18 experts. A pre-test was carried out for adjustments. Then, the simulations were applied and evaluated by 18 medical undergraduate students. They included the participation of 6 pediatric simulated patients aged 9–12 years trained by a drama teacher. According to the results, the scenarios and checklist proved to be valid instruments in content terms (ICV-I > 0.8). The scripts were followed by the simulated pediatric patients, but they had difficulty mimicking a hypoactive state. Some were anxious, but everyone enjoyed participating in the feedback. The simulated parents had difficulty participating and giving space to the child's speech. Participants assessed that the simulations performed as they were proposed and, after experimenting them, felt more prepared. The simulations provided an opportunity for students to practice soft skills by interacting with children in a safe environment. Using children as simulated patients is feasible but presents some challenges. Our study has expanded the ways in which children's health content can be taught. We are investigating whether this training leads to better patient outcomes in real clinical settings.

**Supplementary Information:**

The online version contains supplementary material available at 10.1186/s12909-024-05284-7.

## Background

Children are often marginalized in appointments or excluded from decisions involving their own health. Especially, because they are vulnerable, and the appointments involve the complex triadic relationship patients-parents-physician [1]. However, active participation of children is associated with significant improvements in understanding the health-disease process, adherence to treatment, and emotional health [2]. To medical care effectively, soft skills are essential. In particular, communication, interpersonal skills, rapport and resilience [3].

The national guidelines to medical training emphatically guide a person-centered medical practice [4]. However, interactions with children are often observational [5], while soft skills training is aimed at parents and caregivers [6–8]. This standard differs from the recommended one regarding the autonomy, dignity, and safety of children [9]. In addition, students' first experiences with pediatric patients occur without them being adequately prepared. Therefore, there is a gap to be bridged in medical education [10].

Clinical simulation is a method that allows the creation of credible scenarios for care in a safe environment [11, 12]. In this environment, it is possible to insert pediatric simulated patients, which consist of children trained to consistently portray health conditions [13]. But soft skills scenarios in pediatrics often use adults as simulated patients [6, 8, 14–17]. This is due to many ethical issues involved regarding the risk of maleficence, as well as the challenging logistics for the development of strategies that ensure children are supported and receive adequate training [18].

Physicians also need to develop soft skills to provide care that is focused on the child as part of a family context and to ensure the child's right to be a protagonist in his or her health care [19]. In this context, this study developed, validated, and applied clinical simulation scenarios that promoted the active participation of children. The goal of the study was to teach soft skills in child-centered care to undergraduate medical students. Recommendations for improving simulation scenarios involving simulated pediatric patients were also presented.

## Methods

This is a methodological study to develop three scenarios and a checklist. It was carried out between 18th of July and 10th of November, 2022, during the application of the Child’s Health Module of the Multicampi School of Medical Sciences (EMCM) of the Federal University of Rio Grande do Norte – UFRN, Caicó – Brazil. This module is offered to third-year medical undergraduate students and aims to develop skills to perform clinical examination, diagnosis, and therapeutic management in pediatrics.

### Study protocol

#### Construction of simulation scenarios and evaluator’s checklist

A directed search was conducted with the aim to find materials. Search terms used were: Pediatric medical education AND soft skills OR communication OR interpersonal skills AND simulated patient OR patient simulation AND child OR children. The databases used were Medline/PubMed and Scielo. Books, regulations, articles in English and Portuguese, guides and checklists were considered. But we had trouble finding articles on the subject. So, we did a manual search, contacting some authors for materials.

Based on book chapters [20, 21], EMCM medical course regulations [22] and national guidelines for medical training [4] a preliminary version of the scenarios and checklist was structured. This version was improved based on the Calgary-Cambridge Guide validated in Brazil [23] and with support of a multidisciplinary group composed by 2 physicians with trained in family medicine, 2 psychologists with expertise in health communication and 1 nurse with trained in clinical simulation. They were all professors at EMCM.

The textual elaboration of the scenario followed a pre-established theoretical-practical model from Fabri et al. [24]. The group discussed aspects of soft skills in child-centered care from different professional perspectives. In addition, the psychologists acted to minimize possible harm to the participating children. The same group also participated in the pre-testing phase described below.

#### Validation by judges

The committee was composed of 18 judges, 6 for each scenario. The selection of judges was by convenience. Then, the snowball sampling technique was used, so the professionals who received the instrument acted as key informants, providing the electronic address of three other professionals and so on.

Health professionals with experience in child health, health communication and/or clinical simulation were selected. The criteria for selection of judges were adapted from those proposed by Fehring [25]: master’s degree = 4; master’s degree in one of the cited areas = 1; articles published on the topics = 2; PhD in one of the study areas = 1; clinical experience = 2; teaching experience = 2. A minimum score of 5 points was used.

The judges received a letter of invitation via email, the constructed materials and the validity form via Google Forms®. Each scenario was evaluated regarding comprehensiveness and then item by item concerning clarity and representativeness of the psychometry. For this purpose, a 4-point Likert-type scale was used, as follows: 1 – item not clear or not representative; 2 – item unclear or requiring major revision to be representative; 3 – item quite clear or requiring minor revision to be representative; and 4 – item very clear or representative. There were also spaces throughout the instrument for suggestions and criticism.

To systematize the opinion of experts, the Delphi method was used [26].

#### Pre-test

Nine students from the EMCM/UFRN medicine course participated, selected by purposive sampling. They were regularly enrolled in the course and had already completed the child’s health module. Scenarios were tested and then a session of brainstorm was carried out for semantic evaluation, achievement of objectives, and factors that facilitated or prevented implementation.

The multidisciplinary group of teachers also participated. They made a qualitative analysis of the checklist.

### Application and validation by medical undergraduate students

Eighteen third-year medical students from the EMCM/UFRN who were taking the child health module participated. Exclusion criteria were having already taken the child health module; having already taken more advanced modules of the course; having less than 75% participation in the activities offered; refusal to sign the informed consent form.

They were selected by random sampling. They were then divided into two smaller groups (*n* = 9, each) to experience the simulations. The three scenarios were developed for three consecutive weeks and were applied twice on each application day. Each time, a different student volunteered to realize the appointment, while the others watched the performance in the auditorium via live transmission.

The simulations were designed to last 1 h, intended for: 15 min – briefing; 15 min – scenario; and 30 min—debriefing. The purpose and general objective of the scenarios, the type of simulation, and the sequence of steps until the debriefing were informed in the briefing. Additionally, a fictional contract was established for students to suspend disbelief and acknowledge the scenario.

After simulations, the students anonymously evaluated the experience using a 10-question questionnaire. A Likert-type scale was used, with classification ranging from 1 to 5 in the order of the following concepts: 1 – Totally disagree; 2 – Partially disagree; 3 – Neither agree nor disagree (indifferent); 4 – Partially agree; 5 – Totally agree.

Three professors participated: a psychologist, a physician, and a nurse with training in clinical simulation who led the simulations. There were also the participation of a laboratory technician and a cameraman. The professors received guidance on the objectives and skills to be worked on scenarios, the use of the evaluation instrument, and the debriefing in PEARLS model [27]. They watched the simulations in the control room, which had one-way mirrored glass.

Furthermore, all students involved in the research received study materials, which contained book chapters [20, 21] and videos with examples of communication with children and their families [28, 29]. Then they had expository-dialogued classes on bioethics and pediatrics and soft skills in child-centered care according to development stages.

Six children aged 9–12 years old, who had been previously trained, participated as pediatric simulated patients in both the pre-test and the application. Of these, 2 were boys, 4 were girls, 1 was black and the rest were white. They all belonged to the upper middle class. Each child acted in a single scenario. In turn, two mothers and one father took turns in the roles of simulated parents. All of them were trained by a drama teacher. Simulation facilitator also participated in the training.

### Data analysis

The content validity Index (CVI) was calculated using two mathematical equations: I-CVI (item-level content validity index) and S-CVI/Ave (scale-level content validity index). The I-CVI was used to evaluate the agreement of each item evaluated. It was calculated by adding the number of “3” and “4” responses from the judges or “4” and “5” from the target audience divided by the sum of the total number of responses. The S-CVI/Ave was used to evaluate the mean of the I-CVIs in each domain. It was calculated by summing the I-CVI of all items, dividing by the total number of items evaluated per domain. The I-CVI ≥ 0.8 and S-CVI / ≥ 0.9 were considered desirable for validation [30]. When necessary, the Modified Kappa Coefficient (MKC) was also calculated to assess the possibility of random agreement. An MKC ≥ 0.74 was considered desirable [31].

## Results

### Construction of simulation scenarios and evaluator’s checklist

Each scenario was composed by four domains: 1 – Context and Previous Components; 2 – Scenario Preparation; 3 – Design; and 4 – Final Components. Domains 1, 2, and 4 were identical to three scenarios, while domain 3 addressed the simulated case and specific scripts of each scenario.

The purpose of the scenarios was to teach. Therefore, they were structured with common clinical situations in pediatrics: sore throat and fever, vomiting, and asthma attacks. In addition, the scenarios were organized in increasing levels of complexity and required the performance of different actions to solve the problems (Table 1). The simulations were of the scenic type, set in a medical office for pediatrics, with simulated pediatric patients accompanied by their simulated parents.

**Table 1:** Complexity of the simulation scenarios and problem solution

Scenarios	Somatic problem	Challenge	Problem solution
Scenario 1	Sore throat and fever	The child has pain when swallowing; The child disapproves of pill form or bad tasting medication	Addressing the concerns; Give treatment options; Help the child to become involved in care
Scenario 2	Vomiting	The child disapproves of intravenous injection or administration of saline; Dramatizing child; Rigid parent	To calm down; Recognize feelings and emotions; To establish *rapport;* Inform what the patient has, what will happen and who will perform the care; Encourage the patient to feel in control of the treatment
Scenario 3	Asthma Crisis	Anxious parent; The child is neglecting part of the treatment to demonstrate independence; The child is ashamed of his own state of health; The child is using medication inappropriately; Divergence of information provided by parent and son	To calm down; Recognize feelings and emotions of child; Identify conflicting information and needs To Educate; Help the child solve the problem in order to be healthy and have a normal social life; Encourage the patient to feel in control of the treatment

**Fig. 1:**
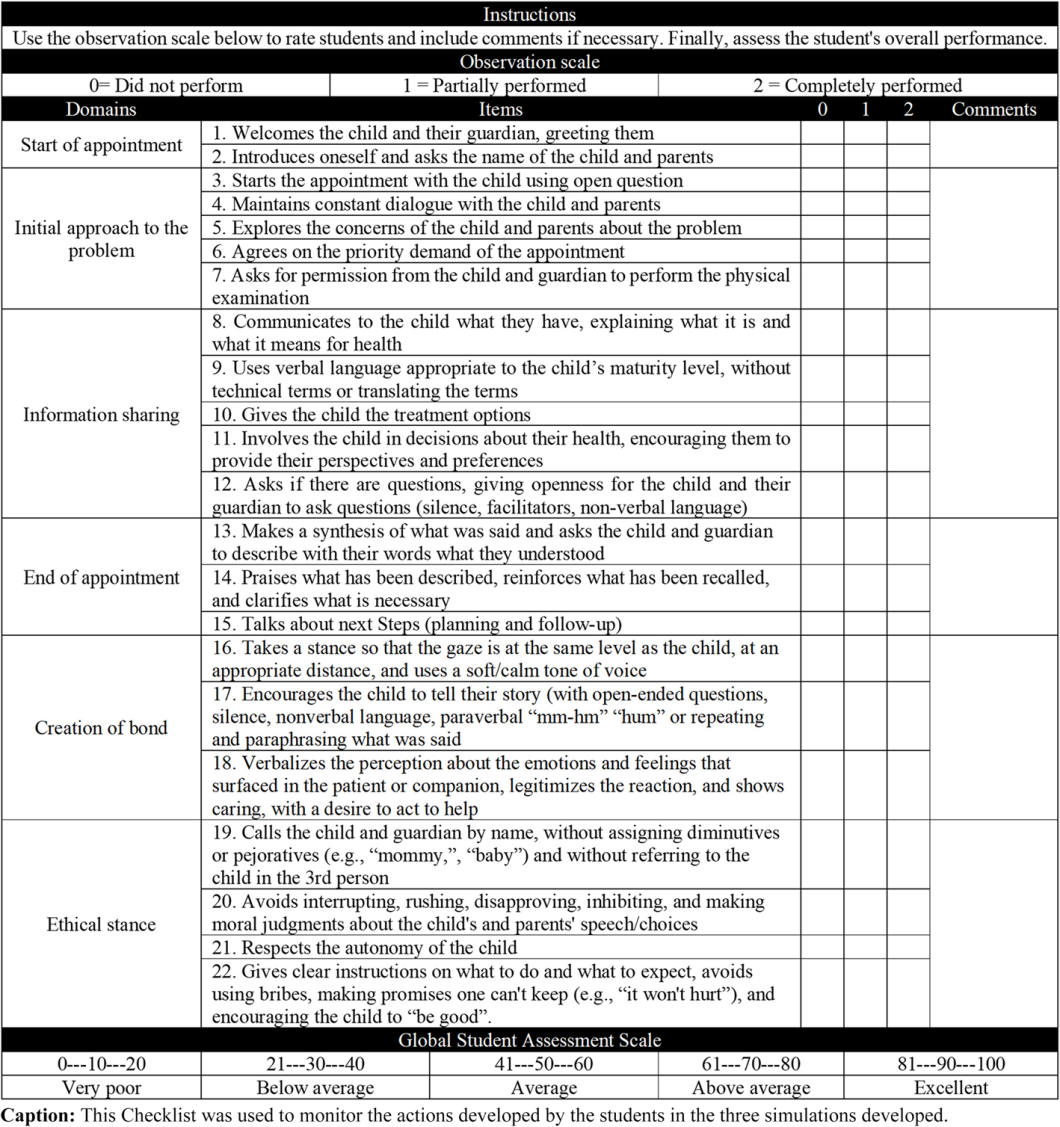
Checklist after content validation and pre-test

In order to monitor the actions developed by the students, a checklist with 22 items divided into six domains was created (Fig. 1). Professors could record whether the candidate "did not perform (0 points), partially performed (1 point), and completely performed (2 points)" the actions, with spaces for comments on each item. There was also a global student rating scale at the end of the checklist.

### Validation by judges

Judges from thirteen different higher education institutions served on the panel. They were predominantly women (16; 89%) and had a mean age of 47 (± 6.8) years old. All of them had at least 10 years of training, teaching experience, and scientific publications in the areas of study. Most had degrees in medicine (8; 44%) and nursing (7; 39%); PhD (16; 89%); theses in the areas of health communication and/or child health and/or clinical simulation (10; 56%); care experience in the areas involved (17; 94%); had as their main activity research, teaching, and extension (16; 89%); and had already given training involving the themes mentioned (17; 94%).

Consensus was reached in the first round of evaluation. Respondents unanimously agreed on the scope of the four domains that make up each scenario (I-CVI = 1.00). It was considered the judgment of the 18 judges for the evaluation of the items identical of the three scenarios (domains 1, 2 and 4) and of the 6 judges for the specific items (domain 3). The I-CVI concerning clarity and representativeness of the items identical to the scenarios exceeded ≥ 0.8 and the CVI/Ave of each domain exceeded ≥ 0.9 (Table 2). In specific items, since there were 6 evaluators, in addition to the CVI, the MKC was also calculated to assess the possibility of random agreement. The respondents were also unanimous (I-CVI and CVI/Ave = 1.00; MKC = 1.00).

**Table 2:** Values of the validation index regarding the clarity and representativeness of domains 1, 2, and 4

	**Clarity**	**Representativeness**
**Items**	**I-CVI**	**I-CVI**
**Domain 1 – context and previous components**		
Theme	1.00	1.00
Target audience	1.00	0.94
Purpose of the simulation	0.94	1.00
Prior knowledge	0.89	1.00
Prior skills	1.00	0.94
Skills and sub-skills to be worked on	0.94	1.00
Activation of prior knowledge	1.00	1.00
**S-CVI/Ave**	0.96	0.98
**Domain 2 – scenario preparation**		
Interventions and expected outcomes	1.00	1.00
Complexity	0.94	1.00
Evaluation method	0.94	0.94
Type of simulation	1.00	1.00
Scenario recording	0.94	0.94
Expected time for the activity	0.94	0.94
Material resources	1.00	1.00
Human resources	1.00	1.00
Physical space/simulated environment	1.00	1.00
Team training for the activity	1.00	1.00
Scenario validation/calibration	0.94	1.00
**S-CVI/Ave**	0.97	0.98
**Domain 4 – final components**		
Scenario development	1.00	1.00
Scene progression	1.00	1.00
Examiner's checklist	0.94	0.94
Debriefing	1.00	1.00
Assessment	1.00	1.00
References	1.00	1.00
** S** **-CVI/Ave**	0.99	0.99

For this evaluation, the number of judges considered was 18. I-CVI – represents the content validity index regarding each evaluated item. S-CVI/Ave is the average of the I-CVIs in each domain. An I-CVI ≥ 0.8. and S-CVI/AVE ≥ 0.9 were considered for validation

The judges made suggestions involving elements of writing, structure, and organization of the materials which were accepted. Some comments also raised concerns about the performance of children as simulated pediatric patients, especially regarding their ability to memorize the lines of the scripts. Furthermore, they pointed out the presence of parents in the scenarios could be a distraction since doctors tend to only communicate with them.

So, general guidelines were inserted with the reasons for simulated pediatric patients and simulated parents speaking or not the information. In the scripts, possible dialogues and their outcomes were inserted, facilitating the orientation of the children. It was explained to the simulated parents that the simulation should prioritize the active participation of the children. Therefore, they should only answer questions directed to them and help the children if they forgot something or asked for help. If for any reason a child felt uncomfortable participating in the simulation, they could ask to go to the restroom and leave the scene (Additional file 1).

Furthermore, the training was structured in a more playful and longer format, with weekly meetings for 2 months. Additional sessions could be scheduled according to the children's needs. The children needed to feel safe and comfortable with the scenarios, the simulation facilitator, and the simulated parents.

### Pre-test

All participants considered the scenarios relevant, well written, capable of achieving the proposed objectives and with adequate time available. Regarding the performance of the simulated patients, it was suggested that the simulated parents should talk and gesture more to denote concern. It was also suggested that the simulated pediatric patients should perform more realistically when using the spray medication during scenario 3 (Additional file 1). Thus, these aspects were reinforced to parents during training, as well as a seal was developed for the spray applicator to prevent children from coming into contact with the medicine when simulating its use.

The evaluators suggested modifying the order of the domains of the checklist (Fig. 1) from “beginning of the appointment, initial approach to the problem, creation of bond, information sharing, ethical stance, and end of the consultation” to “initial approach to the problem, information sharing, end of the consultation, creation of bond, and ethical stance” in order to facilitate filling. In the ethical stance section, they advised modifying the item “respects the autonomy of the child, but recognizes that the parents are responsible for the final decisions of the treatment and involvement of the child” to “respects the autonomy of the child,” since the first option suggests a treatment without partnership.

All child actors participated in the pre-test to experience the simulation process. Some were apprehensive but were reassured by the psychologists and none refused to participate. During the feedback, the children were happy to be involved in medical consultations and to be able to express their preferences.

### Application and validation by medical undergraduate students

The development of the three scenarios occurred according to the scripts, except in Scenario 2. This scenario did not present the expected challenge (Table 1), because the simulated pediatric patients did not present in accordance with the described condition (Additional file 1). They showed a regular general condition that was not consistent with a general state of hypoactivity described in the physical examination. Therefore, the lines associated with the distractor were removed from the script, as well as the information characterizing this condition. The scenario was rerun, and the expected result was verified.

Each of the three scenarios was applied twice and each child acted in a single scenario. In this way, the six actors alternated their participation, so as not to overload each other. The simulated sessions included the direct observation and debriefing. The debriefings sessions were used to discuss positive points and opportunities for improvement. The checklist was used to monitor the actions performed and to work on the students’ self-assessment. The facilitator observed and guided the students' speech, inviting experts and simulated patients to give feedback.

The students evaluated the simulated experiences (Table 3). In all items there was total or partial agreement with the statements, obtaining values ≥ 0.94 (I-CVI) and ≥ 0.98 (S-CVI/Ave). All students (18; 100%) agreed that the simulation was in line with the proposed topic, that a safe and respectful environment was created, that the didactic resources used were appropriate, and that the objectives and goals were explained. They also considered the feedback they received to be positive and stated that they felt more prepared for professional practice after participating in the sessions. Lower proportions of students fully agreed that prior knowledge from previous modules helped them understand the scenarios (11; 61%), felt motivated to participate in the sessions (10; 55.5%), and indicated that the theoretical framework was sufficient to support the solution of the scenarios (9; 50%).

**Table 3:** Evaluation of the simulated experience and values of validation index, according to medical undergraduate students

Item	3	4	5	I-CVI
	**(n) %**	**(n) %**	**(n) %**	
Q1 – Prior knowledge of the previous modules helped in understanding the scenarios	(1) 6	(6) 33	(11) 61	0.94
Q2 – The materials provided for theoretical framework were sufficient to support the solution of the scenarios	(1) 6	(8) 44	(9) 50	0.94
Q3 – The demonstration of support and the procedural support information were essential for the achievement of the objectives	(1) 6	(5) 28	(12) 67	0.94
Q4 – The simulation corresponded to the proposed theme	-	-	(18) 100	1.00
Q5 – A safe and respectful environment has been established	-	(1) 6	(17) 94	1.00
Q6 – The didactic resources used were adequate	-	(4) 24	(13) 76	1.00
Q7 – I felt motivated to take part in the simulation	(1) 5.5	(7) 38.8	(10) 55.5	0.94
Q8 – After the simulations I feel more prepared for the professional exercise	-	(6) 33	(12) 67	1.00
Q9 – I rate as positive the feedback I received during the debriefing	-	(3) 17	(15) 83	1.00
Q10 – The objectives and goals of the simulation were explained in the debriefing	-	(2) 11	(16) 89	1.00
**S-CVI/Ave**				0.98

1 – Totally disagree; 2 – Partially disagree; 3 – Neither agree nor disagree (indifferent); 4 – Partially agree; 5 – Totally agree. I-CVI – represents the content validity index regarding each evaluated item. S-CVI/Ave is the average of the I-CVIs in each domain. An I-CVI ≥ 0.8. and S-CVI/AVE ≥ 0.9 were considered for validation

## Discussion

Physicians’ lack of soft skills for child-centered care is a global challenge [32]. About that, the quality of child health care is compromised [33]. We developed, validated, and applied three scenarios adapted to the active participation of the child. The results showed that our scenarios and checklist are valid tools in terms of content. The simulated pediatric patients acted according to the scripts but had difficulty simulating a hypoactive state. Some were anxious and all enjoyed participating in the feedback. The simulated parents had difficulty participating and giving space to the child's speech. Participants felt that the simulations accomplished what they had proposed, and that they felt more prepared after experiencing them.

Similar scenarios were found in the studies by Frost et al. [6] and Kindratt et al. [8]. However, they focused on parent-centered care. As noted by Wissow et al. [34] and according to the judges who validated the scenarios, physicians tend to show a higher degree of centrality on parents during triadic consultation. Therefore, it is important to have clinical simulation scenarios to teach soft skills in child-centered care to physicians. They need to be validated to verify that they are achieving their intended goals [35] and that there are no elements that could compromise their effectiveness [36].

Content validation followed the previous recommendation of the minimum number of judges [37] and widely used measures were applied to verify agreement [31]. However, our sample was very specialized, with few non-experts, who are also important for evaluating the form, overall impression and categorization of the degree of difficulty [38]. A limiting point was the difficulty of receiving responses in a timely manner.

Judges were also concerned about the use of children as simulated pediatric patients. However, it should be noted that learning soft skills requires the participation of the "other" and cannot be taught using pediatric mannequins [39]. Furthermore, practice with real patients is not recommended, as it disregards patient safety and can cause harmful effects [5]. Therefore, there are important ethical considerations that must be followed. The age of the child (older children have greater autonomy), the role they will play, the duration of the activity, the opportunity for feedback, and a team committed to defending the rights of the child are some of them. Family involvement is essential. Educators must also be sensitive to the child's objection and consent [10, 13, 18].

In the pre-test stage and application stages adjustments were made to actors’ scripts. So, one can note that validation goes beyond the mere evaluation of an instrument and is materialized in practice, with the dynamic interaction of the participants [40]. Moreover, the child actors showed difficulty in maintaining, throughout the scenario 2, a general state of hypoactivity. This aspect had already been pointed out by Khoo et al. [18] and may suggest that children have difficulty staging more specific behaviors. So, it is recommended to work on milder health conditions, as we did, or to include elements in the scenarios that reinforce the seriousness of the situation. For example, placing the child lying on a stretcher, using moulage to simulate some signal, and reinforcing the parents to be more stressed.

Working with simulated pediatric patients presents some potential challenges. The lines in the scripts must be planned, unlike adult simulations, which only require a story line. The simulated parents need to be well oriented so as not to replace the child's speech, but not so rigid that they fail to convey the realism of the situation being experienced. Children need to be familiar with the health conditions covered, as they may feel anxious. A good relationship between the child, the simulated parent and the simulation facilitator is also crucial. Hence the importance of lengthy training and the presence of psychologists on the team.

Our study considered all ethical norms and guidelines recommended for the use of children as simulated patients. The terms of consent and assent were signed; children were over 8 years old; simulations were short; the roles involved everyday situations, which most of them had already experienced; actors were rotated at each simulated session; children could leave the scene if they felt uncomfortable and provide feedback during debriefing.

In addition, among the evaluators there was a psychologist to intervene in possible damage, and all were aligned with the scenario proposal and trained for the debriefing. The debriefing is the most important part of the simulation. It is the basis for fixing and correcting behaviors. It occurs immediately after the simulated experience and is conducted by the students themselves, who analyze the situation and critically reflect on the performance, while the facilitator observes and directs the actions [41].

All students partially or fully agree that the feedback they received after the simulations was positive. They also felt prepared for their professional practice after the experience. This suggests a positive evaluation. Lower proportions of totally agree were obtained about the motivation to participate in the simulations (10; 55.5%) and the theoretical framework being sufficient to support the solution of the scenarios (9; 50%). This can be explained by the fact that our simulations were not focused on highly valued technical skills (hard skills), but on the soft skills that students tend to consider less important [3]. It is noteworthy that the students were at an intermediate level and clinical management of issues was discussed throughout the child’s health module.

It is important to emphasize what may limit the applicability of the research results. Only 6 pediatric patients with similar characteristics participated in the study. The lack of availability of mothers/fathers to participate in the scenarios. The need for teachers with differentiated knowledge and more time to train the children. Regarding the validation process, we did not conduct a new round of validation after incorporating the judges' suggestions.

## Conclusion

Our scenarios and checklist provided an opportunity for medical students to practice soft skills by interacting with children in a safe environment. Using children as simulated patients was feasible, but not without challenges. We recommend working with lighter health situations, including dialogues and possible outcomes in the scripts, allowing more training time and involving the simulation facilitator, familiarizing the child with the simulation process, including psychologists in the team, and valuing the child's feedback in the debriefing.

Despite this, there is a gap in medical education that needs to be discussed. Our study has expanded the ways in which children's health content can be taught in medical curricula. We are now investigating whether this training translates into better patient outcomes in real clinical settings. This research could strengthen the overall robustness and applicability of the study's conclusions.

### Supplementary Information

**Supplementary Material 1. **

## Data Availability

The authors confirm that the data supporting the findings of this study are available within the article and its supplementary materials.
