# Supplementary material for: Validation of clinical simulation scenarios for the teaching of soft skills in child-centered care
Source: BMC Med Educ. 2024 Mar 29;24:355. doi: 10.1186/s12909-024-05284-7 (PMC10981288; doi:10.1186/s12909-024-05284-7)
Supplement: Supplementary file 1 — Supplementary Material 1. [file 12909_2024_5284_MOESM1_ESM.docx]

**Additional file 1 – Scenarios overviews, prompts and scripts**

| **Briefing/Door Command for Medical Learners** |
| --- |
| You will have 20 minutes to complete this station. You are a doctor at the Basic Health Unit and you will see a patient between 9-12 years old accompanied by his/her parents. Use the information you have learned to:   - Perform child-centered medical appointment; - Establish rapport; - Respect the autonomy and dignity of the child; - Show empathy, humanity and solidarity with emotions of patients and their companions. |
| **Guidelines for the pediatric sham patient** |
| - You have to choose a name for the character; - Provide your own data in relation to: sex, gender, age, where you were born and live, religion, where you study and what you like to do; - If they ask you something you don't know or have forgotten, say, "I don't know." Your parent will answer for you; - Avoid making involuntary expressions that may confuse the doctor in relation to the complaint presented, for example, look happy or disdainful; - When the doctor asks for permission to perform the physical examination, you must grant and deliver the paper that we will give you; - If you do not feel comfortable participating in the scenario, say "Mom/Dad, I would like to go to the restroom." |
| **Guidance to simulated mothers/fathers** |
| - You choose a name for the character and provide your own personal data; - When entering the room, show that you are worried, but be collaborative with the conduct of the medical care; - Whenever they ask questions without directing your child, you should answer, but be careful not to dominate the consultation; - Remember, the priority will always be the child's speech and participation. So, if the doctor explains to you the importance of your child's participation, encourage him/her; - If the doctor does not include your child in the consultation, ask the child questions and after hearing his/her responses, answer the doctor; If you forget any information or if the doctor asks something unexpected, say that you don’t know or answer in your own words, without straying from the main idea of the simulation. - If you forget some information, or the doctor asks something unexpected, say that you do not know or answer in your own words, as long as you do not escape the central idea of the simulation; - If your child forgets some information, you should help him/her, but be careful not to dominate the conversation; - When the doctor asks for authorization to perform the physical examination, grant and remind your child to deliver the paper we gave him/her, saying: "Son, where is the paper that the nurse asked you to deliver?"; - If your child says he/she wants to go to the restroom, ask the doctor for leave and accompany him. |
| **Scenario 1: Sore Throat and Fever** |
| For two days you have been experiencing persistent fever, above 38ºC. You have a dry cough, sore throat and difficulty swallowing. The symptoms started after a night of playing with a friend who was sneezing frequently. Your mom/dad is giving you fever-lowering medication and a homemade syrup your grandmother made three times a day. You are very discouraged, refusing to eat solid things. Only juices and water are accepted.  *Role of physical examination:* Regular general condition. Fever of 38.5ºC. Enlarged submandibular, tonsillar, preauricular and cervical lymph nodes. Edema and hyperemia of the tonsils, with the presence of pus. |
| **Scenario 1: Script** |
| **Start of the Consultation** |
| - Make a facial expression that you are not feeling well; - Speak in a husky voice; - Make an expression of pain when swallowing and when coughing. In these moments, direct your hand to your neck. Do this throughout the consultation; |
| **During the Consultation** |
| - If you are asked the reason for the consultation, direct your hand to your neck and say: "My throat is hurting a lot. I can't swallow it right"; - If you are asked what you feel, say, "I feel like I can't swallow. My throat is swollen and it hurts a lot when I cough. It hurts my ear, too." - If you are asked if you have had a fever, say, "Yes." If you are asked how many times, say, "Many times... do you know how many times, mom/dad?"; - If you are asked how it started, say: "It was after the day that I was playing in the street until late at night with my friend"; - If you are asked about having a cough full of secretion, say, "No"; - If you are asked if you are able to eat, say: "Little. I don't feel like it. It hurts the throat"; - If you are asked if you are drinking water, say: "Yes, it is easier to swallow."; - If you are asked if you have allergies, say, "I don't think so"; - If you are asked if you have breathing problems frequently, say, "No"; - If you are asked about medication use, say: "I took a fever medicine that my mother gave me and a syrup that my grandmother made"; - If you are asked how many times you took the medication, say: "Whenever I had a fever my mother gave it to me". |
| **End of the Consultation** |
| - If the doctor prescribes some medication, ask: "Is the medication a pill?" |
| **Outcome 1**   - If the doctor explains to you that it will be a syrup, ask: “Does it taste bad?” - If the doctor says no, accept the treatment. |
| **Outcome 2**   - If the doctor says the medicine is a pill, say: "I would like a syrup." - If the doctor does not change the medication or explain why you have to take the medication in pill form, say: "I don't like to swallow pills." |
| - If you are asked about doubts, say: "When will I be able to ride a bike?" - If the doctor says you will have to return for medical follow-up, say: "Okay." |
| **Scenario 2: Vomit** |
| You're nauseous. You have already vomited 7 times and you are feeling weak. Last night, you snacked on a sandwich that hurt you. You spent the night vomiting, not sleeping properly and could not feed yourself until now. Your mother gave you some tea, but you then threw up. You can't drink juices or water. You had no fever or diarrhea. You came to the health service too afraid of having to take an injection or serum into a vein.  Your mother / father is demanding and if the doctor prescribes an injection or the serum in the vein, she/he will agree and say that if necessary, he will hold you. It will make you very distressed. But if the doctor is empathetic and acts in partnership, reassuring you, your parent will recognize your fear and provide support.  *Role of physical examination:* Poor general condition, with dry mucous membranes, sunken eyes, skin with decreased turgor and elasticity. |
| **Scenario 2: Script** |
| **Start of the Consultation** |
| - Make a facial expression of discouragement; |
| **During the Consultation** |
| - Answer what you are asked with short sentences; - If you are asked what brought you to the appointment, say: "I'm nauseous, vomiting a lot"; - If you are asked what happened, say, "I ate a sandwich and it hurt me." - If you are asked when, say, "Last night on the street."; - If you are asked how you started, say, "Right after I lay down to sleep"; - If you are asked how many times, say: "Many times". - If you are asked what medicine you took, say: “My mother gave me a bitter tea”;   If you are asked what the nausea feels like, say: “I feel like vomiting. I keep thinking I'm going to vomit all the time.”  If you are asked if you feel like vomiting at the moment, say: "No, I just feel nauseous since I stopped eating";  If you are asked if you are able to feed yourself, say "No";  If you are asked if you have had diarrhea, say "No";  If you are asked if you have allergies, say, "I don't know";  If it is explained that the treatment will be an injection or taking serum into the vein look at your mother/father, hold his/her arm and say: "I don't want to, let's go home!" .Show fear;   - If you are asked why, say, "I don't like injections."   If your parents say you have to, say “No! ...I won't!". Start crying or sulking. |
| **Outcome 1**   - If the doctor doesn't reassure you, say, "I want to leave." - Keep crying or looking dumb, without looking at the doctor's face and do not answer any more questions. Keep it up until he reassures you; |
| **Outcome 2**  The first time the doctor tries to explain you why it is important to take the injection or serum into the vein, shake your head negatively and say in a firm tone: "I don't want to!".  If the doctor reassures you, show yourself to be collaborative, look at him and say: "I want to get good, but I'm scared";  When the doctor explains to you how the procedure is and how is the pain you may feel calm down and ask: "Will it hurt a lot?";  Agree with the treatment and say: "I'll let you do it, if it's you";  If the doctor says that it will be someone else who will do it, ask: "Is this person also nice?"; |
| **End of the Consultation** |
| - If you are asked about doubts, say: “No”. - If the doctor says that you will have to admit to take the medication, say: “okay.” |
| **Scenario 3: Asthma Crisis** |
| A month ago you were taken to the pediatrician, because you had been presenting wheezing in the chest that worsened upon contact with dust or animal hair. The pediatrician said you have a disease called asthma and passed a medication to the lung, 3 times a day. At one of these times, you're at school and usually don't take the medication because you're ashamed of your peers. The medication is a spray, which to be released you need to trigger an applicator. You have difficulty using the applicator and inhaling the medication correctly, but you have not accepted help from the teacher. This has hindered treatment. Today, you got sick while playing with your colleagues. You had shortness of breath, chest pain and turned pale. Concerned, the school principal called your mother/father.  At the consultation, you are experiencing slight respiratory fatigue and a feeling of heaviness in your chest. Your mother / father is anxious, afraid that something serious will happen to you. She/he thinks you are taking the medication correctly, because he/she asked the teacher to help you. He/she also does not know that you are ashamed to use the medication in front of your colleagues.  *Role of physical examination:* Regular general condition, eupneic, with decreased breath sounds and diffuse wheezing. Cardiac auscultation with regular rhythm in 2 times, normophonetic sounds, without murmurs. |
| **Scenario 3: Script** |
| **Start of the Consultation** |
| - Pretend to be a little panting. Do this during all the consultation; - Speak giving pauses and with short sentences; |
| **During the Consultation** |
| - If you are asked about the reason for the consultation, say: "I'm short of breath ...   I feel a weight in my chest...";   - If you asked how it started, say, "I was playing running... with my colleagues... and I got sick"; - If you are asked what you felt, say, "I started having shortness of breath... my hands went cold... my eyes darkened... my teacher said I ran out of color;" - If you are asked what was done, say, "My teacher sat me down and ... then she took my medicine and helped me to take...; - If you are asked how long it has been since you took the medication, say, "It was close to the time my mom/dad arrived"; - If you are asked if this has happened other times, say, "No, the other times... I just had a wheeze in my chest and... cough"; - If you are asked what you did, say, "I don't remember... but when I went to the doctor... he passed the medicine and I got better."; - If you are asked what medicine it is, say: "It is a spray… that I inhale three times a day … in the morning, afternoon, and night." - If you are asked if you use the 3 times, say, "When I'm at school... I don't like to use it"; - If you are asked if you had already taken the medication today, before you got sick at school, say: "No ..."; - If you are asked if you always do this, say, "Sometimes..."; - If you are asked why, say, "I'm embarrassed... my colleagues keep looking."; - If you are asked why you feel ashamed, say, "Because everyone keeps looking at me... I keep thinking... Why only do I have to use this spray? ... it seems that only I have a problem."; - If you are asked if your colleagues know what the medication is for or if you have already talked to them about it, say: "No."; - If you are asked if your teacher helps you take your medication at school, say, "No... I take it alone"; - If the doctor asks you to demonstrate how you use the spray, do not remove the seal that was placed on the applicator and demonstrate the use in the wrong way, according to the training; - When the doctor is teaching you how to use the spray correctly, suck the medication the wrong way for 2 times; - If you are asked if you have been feeling tired when doing your activities at home and when playing, say "I think so...".; - If you are asked if you have slept well, say, "I sleep well"; - If you are asked about having a cough full of secretion, say "No"; - If you are asked about the presence of allergies, say: "Yes, the dust and hair of the neighbor's cat"; - If you are asked if your home has carpets, curtains and pets, say: "No"; - If you are asked about using other medications, say, "No"; |
| **Outcome 1**   - If the doctor does not explain why it is important to use the medication every day and at the correct times, say in a firm tone: "You just tell me to use this spray! ... I'm tired of having to use this every day!" - If you are asked if you know what can happen if you don't take the medication correctly, say "I'll be tired forever!" - If the doctor does not take your complaint seriously and does not explore possible solutions, say: "I do not want to use this!" |
| **Outcome 2**   - If the doctor takes your complaint seriously and tries to find a solution, be cooperative. - If you are asked if there is any way for you to feel more comfortable using the medication, say: "Maybe if I could leave the class... and go somewhere where nobody could see me." |
| **End of the Consultation** |
| - If you are asked about doubts, say: “No”. - If the doctor says that you will have to admit to take the medication, say: “okay.” |
